# Identifying Autism Gaze Patterns in Five-Second Data Records

**DOI:** 10.3390/diagnostics14101047

**Published:** 2024-05-18

**Authors:** Pedro Lencastre, Maryam Lotfigolian, Pedro G. Lind

**Affiliations:** 1 Department of Computer Science, Oslo Metropolitan University, N-0130 Oslo, Norwaypedrolin@oslomet.no (P.G.L.); 2 OsloMet Artificial Intelligence Lab, Pilestredet 52, N-0166 Oslo, Norway; 3 NordSTAR—Nordic Center for Sustainable and Trustworthy AI Research, Pilestredet 52, N-0166 Oslo, Norway; 4 Simula Research Laboratory, Numerical Analysis and Scientific Computing, N-0164 Oslo, Norway

**Keywords:** autism, autism diagnosis, eye-tracking, eye gaze dynamics, intelligent health, AI

## Abstract

One of the most challenging problems when diagnosing autism spectrum disorder (ASD) is the need for long sets of data. Collecting data during such long periods is challenging, particularly when dealing with children. This challenge motivates the investigation of possible classifiers of ASD that do not need such long data sets. In this paper, we use eye-tracking data sets covering only 5 s and introduce one metric able to distinguish between ASD and typically developed (TD) gaze patterns based on such short time-series and compare it with two benchmarks, one using the traditional eye-tracking metrics and one state-of-the-art AI classifier. Although the data can only track possible disorders in visual attention and our approach is not a substitute to medical diagnosis, we find that our newly introduced metric can achieve an accuracy of 93% in classifying eye gaze trajectories from children with ASD surpassing both benchmarks while needing fewer data. The classification accuracy of our method, using a 5 s data series, performs better than the standard metrics in eye-tracking and is at the level of the best AI benchmarks, even when these are trained with longer time series. We also discuss the advantages and limitations of our method in comparison with the state of the art: besides needing a low amount of data, this method is a simple, understandable, and straightforward criterion to apply, which often contrasts with “black box” AI methods.

## 1. Introduction

Autism spectrum disorder (ASD) is a neurodevelopmental disorder for which typical symptoms include deficits in reciprocal social communication (both verbal and non-verbal), interaction, and behavior, sometimes marked by stereotypic body movements, rigid routines, and extreme (high or low) activity connected to sensory input. Being a spectrum disorder, it can manifest very differently from person to person. Consequently, the diagnosis ASD usually revolves around cognitive tests that could require hours of medical examinations and implies several challenges. The amount of time it takes to diagnose this condition makes diagnosing it a resource-consuming endeavor. Because of this, despite the impact that the condition has on someone’s life, it is believed that two in three people who fall within the criteria of ASD are diagnosed in adulthood [1].

Though focusing essentially on visual attention features, eye-tracking has arisen as a convenient method to give an indication of a diagnosis of autism at an early age, some as young as 10 months old [2]. It has been shown that not only do individuals with ASD have different interests and thus look at different areas in images [3,4,5] but their eye movements have different dynamical properties [6,7].

Studies relying on different Areas of Interest (AoIs) among ASD children and Typically Developed (TD) ones often rely on the fact that ASD individuals are commonly more interested in geometric objects and non-organic movements as opposed to animal or human shapes [3,4]. In this paradigm, several images are shown, typically containing a biological element, such as a human fact, and a non-biological element, such as a daily object. The amount of time one individual spends looking at each element in an image is then counted and, based on it, a suggestion for ASD diagnosis is produced. Using this approach, past studies have been able to identify individuals with ASD with an accuracy of around 85% [5].

Analyses of the dynamics of gaze trajectories can result in good classification accuracy and, while it does not give the behavioral insights of the AoI analyses, they can shine a light on brain functions related to ASD. An example is the study linking lower saccadic acceleration and peak velocity to dysfunctions in the cerebellum and brainstem [6]. Further differences have been reported with ASD children having higher average saccadic velocities [7] as well as extended saccadic latencies and increased difficulties in smooth pursuit tasks [8].

Some hybrid methods considering both an AoI analysis and dynamics of gaze trajectories have been applied to highly functioning autistic adults, and it has been argued that this group typically explores more web pages when interacting with them and retrieves different information [9]. Using a metric of how exploratory an individual is when looking at an image, these studies provide a method that can detect whether an individual has ASD with an accuracy of around 75% [10].

Regarding the classification task of gaze trajectories originating from ASD or TD children, AI algorithms have been enjoying recent success [11]. When applied to eye-tracking data, these algorithms have consistently provided classification accuracy values over 90% [12,13,14]. Nonetheless, despite their remarkable success, these methods still yield caution when assisting doctors in diagnosing this condition. Typically, AI raises questions of reproducibility since it is usually unclear how the algorithms come to specific outputs.

In this study, we introduce a new quantity related to how exploratory an individual is for the first 5 s after encountering an image. With a classification accuracy above 90%, we show that this quantity outperforms the usual eye-tracking metrics regarding fixations and saccades as well as some of the most modern AI algorithms.

## 2. Data and Methods

Our data set was collected by the authors in Ref. [15]. It is comprised of data from 59 children with ages ranging from 3 to 12 years old, both male and female. Of these 59 files, 2 were empty and from the remaining 57, 28 (age 7.74±2.73, 24 male) files corresponded to ASD children, and the remaining 29 were obtained from TD children (age 8.02±2.89, 13 male). The ASD diagnosis was confirmed by health professionals using standardized tools (Autism Diagnostic Interview-Revised [ADI-R], and Autism Diagnostic Observation Schedule–Generic [ADOS-G]). Participants in the ASD group were evaluated in multidisciplinary specialty clinics. Psychologists assessed the severity of autism using the CARS evaluation [16] and the Early Social Communication Scale [17].

Each participant sat at around 60 cm away from a 1280 × 1080 monitor. The SMI Red-M eye-tracker with a 60 Hz sampling rate was used, and only right-eye data were provided. Each participant performed several trials that were composed by watching an image or video. The images portrayed an actress pointing towards a non-organic distractor of a similar size to the actress’ head (a balloon, for example). In videos, the scene started with a handwaving cartoon. Afterward, the woman in the video greeted the child with a friendly ’Hello, how are you?’ before directing their attention, either through gaze, speech, gesture, or a combination thereof, towards the non-organic element of the scene. Children with ASD performed, on average, 5.5 trials (154 total), while the TD ones performed 7.8 trials on average (225 total).

**Figure 1:**
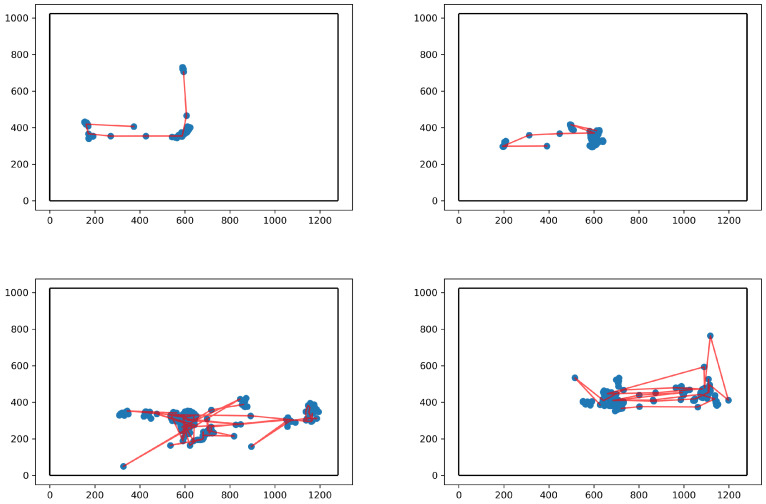
Illustration of the first 300 data points (corresponding to 5 s) from four gaze trajectories, two of them belonging to TD children (**top**) and two to children with ASD (**bottom**). All gaze points are marked as blue circumferences, while the parts of the saccade labeled gaze trajectory are represented as red lines. The borders of the computer screen at which the children were looking are marked with black lines.

Each trial has a corresponding eye-tracker file, composed of information about the time-stamp a sample was collected, the left and right eye gaze positions, a classification of whether a given data point belonged to a saccade or fixation, and the pupil diameter of both left and right eye measured in millimeters. Here, we focus on the gaze position in the screen (see Figure 1) and we convert from the eye-tracker’s native unit of pixels to degrees of visual angle, i.e., the angle between the gaze and the normal to the screen plane.

### 2.1. Benchmark1: Standard Eye-Tracking Metrics

**Figure 2:**
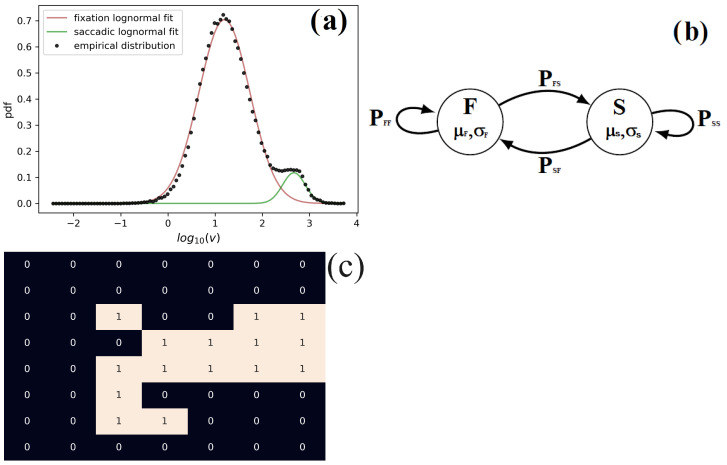
(**a**) Log-velocity distribution of TD children and children with ASD. (**b**) Illustration of a 2-state process where the states F (Fixation) and S (Saccade) are characterized by the averages $\mu_{F}$ and $\mu_{S}$ and standard deviations $\sigma_{F}$ and $\sigma_{S}$, respectively, corresponding to the log-velocity of fixations (respectively, saccades). These states alternate throughout time with probabilities $P_{FS}$ if the transition is from saccade to fixation, or $P_{SF}$ if from fixation to saccade. (**c**) Illustration of the visited area *A*. The areas marked with “1” represent the areas of the image visited in the considered time period. In this case, *A* was calculated for the first 300 data points and, for illustration purposes, the area of each grid’s square was enhanced to around 10 degrees of visual angle in each axis.

The first model we use to classify ASD gaze trajectories uses six metrics relative to standard dynamical fixation/saccade quantities. To compute them, we define the eye gaze velocity as

(1) $$v\left(t_{i}\right)=\frac{\sqrt{{\left(X\left(t_{i}\right)-X\left(t_{i-1}\right)\right)}^{2}+{\left(Y\left(t_{i}\right)-Y\left(t_{i-1}\right)\right)}^{2}}}{t_{i}-t_{i-1}}\;,$$

where *X* and *Y* are the horizontal and vertical gaze spatial coordinates and *t* the time in seconds of each gaze recording. As shown in Figure 2a the velocity is log-normal distributed and that fixation and saccades present different typical velocities.

Noticing the log-normality of the velocity, we use henceforth the logarithm of the velocity in Equation (1) to define six additional quantities, namely, the following:

- The average of the log-velocity $\mu_{F}$ over the $N_{F}$ data points that are labeled as fixations:
  (2) $$\mu_{F}=\frac{\sum_{1}^{N_{F}}\log\left(v(t)\right)}{N_{F}}\;.$$
- The standard deviation of the log-velocity $\sigma_{F}$ over the $N_{F}$ data points that are labeled as fixations:
  (3) $$\sigma_{F}=\frac{\sqrt{\sum_{1}^{N_{F}}{\left(\log\left(v(t)\right)-\mu_{F}\right)}^{2}}}{N_{F}}\;.$$
- The average of the log-velocity $\mu_{S}$ over the $N_{S}$ data points that are labeled as fixations:
  (4) $$\mu_{S}=\frac{\sum_{1}^{N_{S}}\log\left(v(t)\right)}{N_{S}}\;.$$
- The standard deviation of the log-velocity $\sigma_{S}$ over the $N_{S}$ data points that are labeled as saccades:
  (5) $$\sigma_{S}=\frac{\sqrt{\sum_{1}^{N_{S}}\log\left(v(t)\right)-\mu_{S}{)}^{2}}}{N_{S}}\;.$$
- The probability $P_{FS}$ of the saccade-labeled point following a fixation labeled one:
- The probability of a fixation-labeled point following a saccade-labeled one: $P_{SF}$.

Figure 2b illustrates the relationship between these quantities and illustrates a two-state Markov system [18].

Several algorithms exist to classify saccades and fixations [19]. Here, for simplicity, we will use the classification that is provided in the existing data files which are created according to the eye-tracker’s manufacturer algorithm [20].

To create the classification model, we use the three quantities that are statistically significant according to Table 1, namely, $\mu_{F}$, $\sigma_{S}$, and $P_{FS}$. With these variables, a logistic model of classification is defined by:

(6) $$p\left(\mu_{F},\sigma_{S},P_{FS}\right)=\frac{1}{1+\exp\left(-L\right)}$$

with L representing a linear regression of the chosen quantities, namely,

(7) $$L=\beta_{0}+\beta_{\mu_{F}}\mu_{F}+\beta_{\sigma_{S}}\sigma_{S}+\beta_{P_{FS}}P_{FS}\;,$$

where $p(\mu_{F},\sigma_{S},P_{FS})$ is the model assessment of the probability that a participant with estimated parameters $\left(\mu_{F},\sigma_{S},P_{FS}\right)$ belongs to the ASD group. The β coefficient is estimated computationally by minimizing the logistic loss, i.e., the negative log-likelihood.

**Table 1:** Mean of each parameter plotted in Figure 3 for both ASD and TD groups alongside the *p*-value of the Student’s *t* test. From this table, it is clear that $\mu_{F}$, $\sigma_{S}$, $P_{FS}$ and Λ are statistically significant at 95% confidence level. It is unclear if $\mu_{S}$ is different between groups, and no difference was found in $\sigma_{S}$ and $P_{SF}$.

Param.	Mean TD	Mean ASD	*t* Test
$\mu_{F}$	1.04	1.20	$4.3\times 10^{-3}$
$\sigma_{F}$	$4.37\times 10^{-1}$	$4.29\times 10^{-1}$	$3.1\times 10^{-1}$
$\mu_{S}$	2.13	2.16	$3.2\times 10^{-2}$
$\sigma_{S}$	$7.08\times 10^{-1}$	$6.21\times 10^{-1}$	$1.2\times 10^{-4}$
$P_{\mathrm{FS}}$	$5.02\times 10^{-2}$	$6.17\times 10^{-2}$	$2.2\times 10^{-2}$
$P_{SF}$	$3.58\times 10^{-1}$	$3.25\times 10^{-1}$	$2.7\times 10^{-1}$
Λ	32.8	36.2	$9.4\times 10^{-12}$

### 2.2. Benchmark2: AI Classification Algorithm

To create an AI benchmark, we use one of the state-of-the-art algorithms for time-series classification: the InceptionTime architecture [21] following the implementation in the python tsai library [22]. This algorithm is implemented for time-series classification following its success in image classification tasks. With a primary focus on capturing diverse temporal features across multiple scales, InceptionTime employs a distinctive Inception Module, reminiscent of its image-centric counterpart.

The Inception Module is a crucial component, featuring parallel convolutional layers with varying filter sizes. This allows the model to select and extract temporal patterns at different resolutions at the same time, enhancing its ability to deal with statistical features at different time scales. To reduce computational costs, InceptionTime integrates bottleneck layers, employing 1 × 1 convolutions to reduce channel dimensions before engaging in more computationally intensive 3 × 3, or 5 × 5 convolutions, before finally employing a fully connected layer.

A key strength of InceptionTime lies in its incorporation of shortcut connections, addressing the notorious vanishing gradient problem. These connections facilitate smoother gradient flow during training, contributing to the successful training of deep networks.

In the architectural culmination, fully connected layers and output layers are strategically positioned for final classification. The resulting model demonstrates a proficient capacity to discern intricate temporal patterns, making it particularly well suited for diverse time-series classification tasks.

### 2.3. A New 5 s Exploratory Metric

Finally, in contrast with benchmark1, a quantity is defined which is not based on the eye gaze velocity, but, instead, it measures how exploratory a gaze trajectory is. More precisely, we consider the target screen as a spatial grid of squares of length 0.5 degrees of visual angle and then quantify the number of squares a gaze trajectory visits in a given time period τ. We call this quantity the “visited area” *A*—see the bottom panel of Figure 2. For statistical reasons, we consider the average visited area per unit of time, namely,

(8) $$\Lambda \left(\tau ,N\right)=\frac{1}{N}\sum_{k=0}^{N}\frac{A(k\tau )}{k\tau }\;,$$

with τ=1/60 s and N=300, corresponding to the first 5 s of data.

This metric can be seen as a weighted average of how exploratory a gaze trajectory is, with the first data points having an added weight on this metric. To establish a classification based on it, a threshold is defined so as to maximize the training set accuracy. In all models considered here, a 5-fold cross-validation is used instead of a single train–test split. This not only maximizes the usage of the data at our disposal but makes the results less dependent on a particular choice of training and testing subsets. For our analysis, we choose the values τ=0.167 s, and N=300. This corresponds to the number of newly visited areas per second, averaged over time windows ranging from 0.167 s to 5 s.

## 3. Results

We apply the previously described classification model to each individual trial in our data set, given a minimum of 500 data points excluding blinks. We observe in Figure 4 that the Γ metric is statistically significant across groups, even when fewer data are considered. We have chosen 5 s of data, as it creates a good compromise between the fluctuations of this metric and the constraints of gathering large amounts of data, but, arguably, a smaller amount of data could have been used.

**Figure 3:**
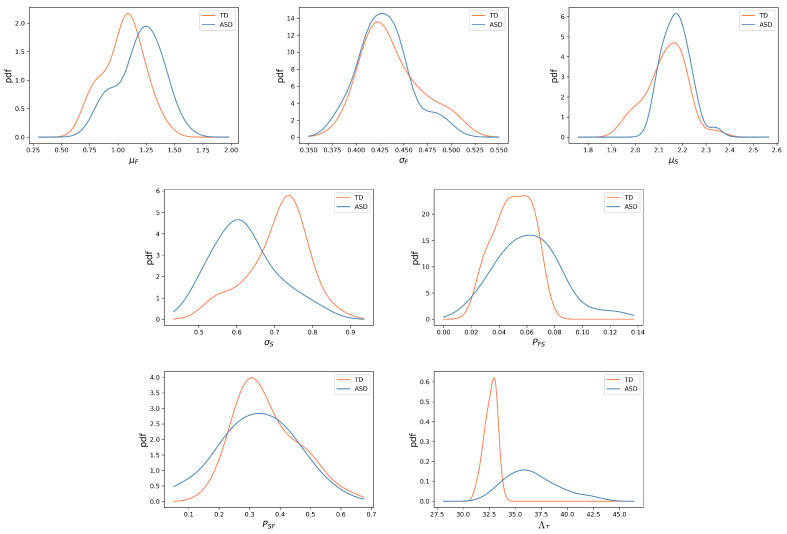
Probability density functions (pdf) for each quantity analyzed here for ASD (blue) and TD (orange) children. In the top row, we have the pdfs of the fixation and saccade parameters ($\mu_{F}$,$\sigma_{F}$, $\mu_{S}$,$\sigma_{S}$) across all participants. On the bottom row, we have the pdfs of the parameters $P_{FS}$, $P_{SF}$, and Λ. From visual inspection, we observe that this latter variable is the one where the difference in distribution is the starkest. This can be verified in Table 1.

**Figure 4:**
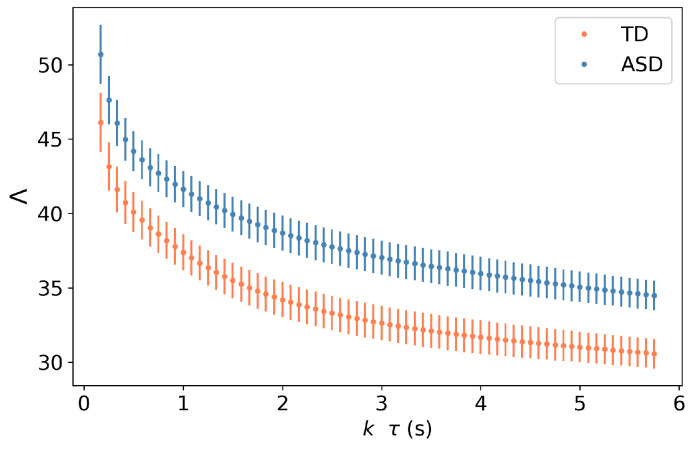
Values of Λ(τ,N) for different values of NΔτ, corresponding to the amount of time considered to compute this metric. We observe that for different multiples of τ, the values of Λ are statistically significant across groups. The symbols indicate the average value of Γ over all participants, and the error bars represent the corresponding standard deviation.

In Figure 3, we plot the distribution of the seven previously mentioned parameters for children with ASD and TD children (six of them concerning dynamics of fixations and saccades, as well as our newly introduced metric) and in Table 1, we show the typical value of each of these quantities for each group alongside two statistical tests to verify if these can be considered different: the Mann–Whitney–Wilcoxon (MWW) statistical test and the Student’s *t* test. While the MWW test is not directly concerned with differences in average values, it has as a null hypothesis that, if one randomly selects participants from two groups, the values of a given parameter in one of these populations are consistently higher (or lower) than the other. This test has the advantage of not requiring a normality assumption and thus, injective non-linear transformations of variables have no impact on this statistic, while the same is not true for the Student’s *t* test.

An analysis of the different parameters shows significant differences between groups. Namely, we see that fixations are typically faster in children with ASD, even though there is significant overlap between groups in this quantity (Figure 3). Fixations, however, appear to be equally dispersed for TD and ASD children, with the small difference in distributions and mean values appearing not statistically relevant according to the MWW test and the *t* test.

When it comes to saccades, we see the converse picture, with their dispersion being statistically significant, with the saccades from TD children being more dispersed, while the ones from children with ASD are more concentrated around the mean. Whether the distribution of log-velocity of saccades is the same in the two groups is not clear, with the *p*-value of the MWM test and *t* test being 0.067 and 0.032, respectively.

Looking at $P_{FS}$ and $P_{SF}$, we notice also different dynamical behaviors, namely in $P_{FS}$, which indicates that ASD children have more frequent saccades and, consequently, shorter fixations. However, the fact that the difference in $P_{SF}$ does not appear to be significant indicates that the average length of saccades is statistically the same between groups.

The difference in $P_{FS}$ may help us understand the very substantial difference we see in the values of Λ, showing that children with ASD explore more of an image when first encountering it. Indeed, from visual inspection, it is clear that this quantity is the one with the least amount of overlap between groups and thus, the one that better distinguishes between groups, with *p*-values of $1.5\times 10^{-8}$ and $4.2\times 10^{-9}$ for the MWW and *t* test, respectively. Distinguishing ASD and TD gaze trajectories from these quantities is the subject of the next subsection.

The relative importance of each normalized parameter is proportional to the absolute value of its corresponding β coefficient. In this case, we observe that $P_{FS}$ is the variable most impacting the regression, with $\beta_{P_{FS}}=18.5$, followed by $\beta_{\mu_{F}}=16.3$ and $\beta_{\sigma_{s}}=0.6$. An analysis of the β coefficients is complemented by the permutation feature importance PI, which is calculated by permutating a single variable value and evaluating the decrease in its score [23]. The more the score of the model is affected by permutating one of the variable’s values, the higher the importance of that variable. Here, again, we observe that feature importance is greater for $P_{FS}$ with $PI_{P_{FS}}=0.23$ followed by $PI_{\mu_{F}}=0.18$ and $PI_{\sigma_{S}}=0.05$.

In the second benchmark model, we use an inception time neural network. In our implementation, we start by normalizing the variable that we feed to the neural network (log(v)) so that its range is between 0 and 1. Regarding the hyperparameter of our model, we use a batch size of 64 samples and an initial learning rate of $10^{-3}$, which is then optimized using the Adam optimizer [24].

**Figure 5:**
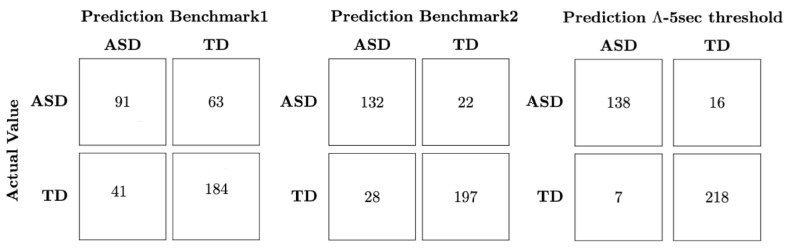
Confusion matrices relative to the classification results for the two benchmark models as well as the classification using the Λ metric. Besides needing fewer data, we observe that the quantity Λ has a higher accuracy 93.9% compared to 72.5% and 86.8% of benchmark1 and benchmark2 respectively. Relative to the other measures, the Λ classification also outperforms the benchmarks, with sensitivity, specificity and precision values of 95.1%, 93.1% and 89.6% respectively, compared to the values of 68.9%74.4%, 59.0% for the same metrics for benchmark1 and 82.5%89.9%, 85.7% for benchmark2.

Finally, in using the Λ metric, we need to find the threshold that can best distinguish both groups. For this task, we used logistic regression, but a comprehensive search across Λ values can be employed. We see in Figure 5 that the Λ metric provides a better classification accuracy (93.9%) than both benchmark1 (72.5%) and bechmarkk2 (86.6%). The better performance of the Λ-based classification model extends to the other classification measures, such as sensitivity, specificity, and precision.

## 4. Discussion

In this work, we provide a novel classification procedure to distinguish ASD from TD children, and investigate the differences in the dynamics of gaze trajectories. Firstly, we observe in this data set that fixations are on average faster for ASD children ($\mu_{F}$) and shorter in duration ($P_{FS}$). This contrasts with a previous study reporting that fixation durations were the same for children with ASD [25] but are in line with a previous study on infants as young as 6 months old [26]. This can help explain how children with ASD explore an image more. However, these two metrics do not distinguish between groups as well as the Λ metric. These fixation dynamics imply that it is also harder to separate fixations from saccades in the ASD group.

We also observe that children with ASD do not differ from children with TD in their average velocity ($\mu_{S}$) or in their duration ($P_{SF}$). Due to the lower value of $\sigma_{S}$ in children with ASD, it is likely that the saccade peak velocity (meaning the maximum velocity in a saccade) is lower for this group. Saccade peak velocity is, however, a quantity more susceptible to measurement noise than the average velocity since it considers only one data point per saccade.

These results on saccades contrast with others mentioned in the literature, namely, the ones from Ref. [27], which indicates slower and shorter saccades for ASD children, which, when taken in isolation, would indicate decreased exploration of images by children with ASD. The study from Ref. [27] presents a ROC-AUC between 0.670 and 0.733.

Three points should be noted. First, the number of participants in the available data set is limited (57 participants with 379 trials) and is not gender balanced in the ASD group, with a majority of male participants. Second, our analysis was performed always using the first five seconds of each trial set. When we consider any other five-second window of the trials’ trajectory, we find lower accuracy levels of the Λ-based classification model, namely, around 70%. In this scope, we also point to reference [28], which gives evidence that gaze dynamics is more exploratory during the first seconds than in the rest of the trajectory. In particular, the first 3 s are characterized by larger and longer saccades with shorter fixations. Third, while our results provide evidence that children with ASD may explore images more, it is unclear what features of ASD are at the origin of this effect. It is reported that autistic individuals have a larger propensity to avoid human faces and typically focus more on geometrical patterns, which could create a need to explore an image more when encountering it. Nonetheless, further studies are needed to clarify which ASD features influence the difference in the Λ metric between TD and ASD children.

The Λ metric can inform clinical decisions without significantly extending the already lengthy ASD diagnosis process, as it requires only small amounts of data. This might become increasingly relevant in the near future, as methods of tracking the eyes using regular computer/phone cameras are being developed [29]. Furthermore, besides its accuracy and the need for fewer data, this metric is not dependent on definitions of fixations and saccades, which is still a matter of debate among researchers, and for which several competing algorithms exist [30,31]. The Λ metric is simple to conceptualize and implement, and it offers a clear, explainable approach in contrast to the often opaque AI-driven methods [32].

## 5. Conclusions

The main contribution of our work is the introduction of a new metric that efficiently distinguishes children with ASD from TD children that needs only 5 s of eye-tracker data and which has a clear interpretation. This criterion provides accuracy in the range of the most recent AI models [12,13,14]. We introduce a new metric Λ, quantifying how exploratory individuals are when encountering images and which relies on only 5 s of eye-tracking data. This metric is able to detect ASD in children with an accuracy of 93.9%, surpassing the classification accuracy of classification models using the usual metrics of fixations and saccades (accuracy 72.5%) and state-of-the-art AI classifier (89.6%). Therefore, our metric provides accuracy in the range of the most recent AI models [12,13,14], with the advantage of being understandable. We discuss how this enhanced exploratory behavior might be reflected in some parameters of the usual fixation/saccade models, namely, with faster fixations and more frequent saccades.

This study provides a new metric that can assist in diagnosing ASD and can help in the development of user-friendly, transparent, and reproducible classification techniques of ASD gaze trajectories. As the global community continues to recognize the significance of early and accurate ASD diagnosis, it is our hope that this research will drive advancements in both technology and practice, ensuring better outcomes for those on the spectrum. 

## Data Availability

This article uses publicly available data which are appropriately cited in the manuscript.

## Abbreviations

The following abbreviations are used in this manuscript:

ASD	Autism Spectrum Disorder
TD	Typically developed
